# Editorial

**Published:** 1855-04

**Authors:** 


### THE MEDICAL EXAMINER.

### PHILADELPHIA, APRIL, 1855.

### AMERICAN MEDICAL ASSOCIATION.

The eighth annual session of the American Medical Association will shortly be held in this city. We sincerely hope that the assembled delegation will prove a numerous one, and that every part of the Union will be represented. We can assure them, whatever be their number, that they will be heartily welcomed, and that no efforts will be spared on the part of Philadelphia, to render their short sojourn in it as agreeable to them as it is in the power of its physicians to make it.

As the great centre of medical education, from which so many of the wise and good of our profession have gone forth to their distant fields of duty, Philadelphia must ever be regarded with interest by the medical corps throughout the country. The spot in which the first seeds were sown, from which so numerous a band is now reaping the fruits, in an honorable, if not lucrative harvest, cannot but have a warm place in their affections. The coming occasion will afford so favorable an opportunity to its *alumni* of once more revisiting the *alma mater* of their youth, that very many of them, we trust, will avail themselves of it.

The vast interests which are embarked in the Association—the magnitude of the stake for which it was created—the full organization and complete reform of the profession—are considerations unnecessary here to dwell upon. We shall only observe that its influence in elevating the medical character and intelligence of the country, and in cementing those bonds which should unite us as one body, is already widely felt. Nor can we permit ourselves to doubt for a moment, should it still continue to be conducted in the proper spirit, that all its objects will be ultimately secured to the profession.

Rejoicing in the opportunity once more afforded us of reciprocating the warm and generous courtesies, so frequently extended to the profession of our city, we sincerely trust that the anticipated meeting will prove, in every respect, a satisfactory one.

